# Identification of long intergenic non-coding RNAs (lincRNAs) deregulated in gastrointestinal stromal tumors (GISTs)

**DOI:** 10.1371/journal.pone.0209342

**Published:** 2018-12-17

**Authors:** Ugne Gyvyte, Juozas Kupcinskas, Simonas Juzenas, Ruta Inciuraite, Lina Poskiene, Violeta Salteniene, Alexander Link, Matteo Fassan, Andre Franke, Limas Kupcinskas, Jurgita Skieceviciene

**Affiliations:** 1 Institute for Digestive Research, Academy of Medicine, Lithuanian University of Health Sciences, Kaunas, Lithuania; 2 Department of Gastroenterology, Academy of Medicine, Lithuanian University of Health Sciences, Kaunas, Lithuania; 3 Institute of Clinical Molecular Biology, Christian-Albrechts University, Kiel, Germany; 4 Department of Pathological Anatomy, Academy of Medicine, Lithuanian University of Health Sciences, Kaunas, Lithuania; 5 Department of Gastroenterology, Hepatology and Infectious Diseases, Otto-von-Guericke University, Magdeburg, Germany; 6 Department of Medicine (DMID), Surgical Pathology & Cytopathology Unit, University of Padua, Padua, Italy; University of South Alabama Mitchell Cancer Institute, UNITED STATES

## Abstract

Long intergenic non-coding RNAs (lincRNAs) are >200 nucleotides long non-coding RNAs, which have been shown to be implicated in carcinogenic processes by interacting with cancer associated genes or other non-coding RNAs. However, their role in development of rare gastrointestinal stromal tumors (GISTs) is barely investigated. Therefore, the aim of this study was to define lincRNAs deregulated in GIST and find new GIST-lincRNA associations. Next-generation sequencing data of paired GIST and adjacent tissue samples from 15 patients were subjected to a web-based lincRNA analysis. Three deregulated lincRNAs (*MALAT1*, *H19* and *FENDRR*; adjusted p-value < 0.05) were selected for expression validation in a larger group of patients (n = 22) by RT-qPCR method. However, only *H19* and *FENDRR* showed significant upregulation in the validation cohort (adjusted p < 0.05). Further, we performed correlation analyses between expression levels of deregulated lincRNAs and GIST-associated oncogenes or GIST deregulated microRNAs. We found high positive correlations between expression of *H19* and known GIST related oncogene *ETV1*, and between *H19* and miR-455-3p. These findings expand the knowledge on lincRNAs deregulated in GIST and may be an important resource for the future studies investigating lincRNAs functionally relevant to GIST carcinogenesis.

## Introduction

Gastrointestinal stromal tumors (GISTs) are the most common mesenchymal (non-epithelial) tumors of the gastrointestinal tract, with a majority of cases localized in stomach (55.6%) and small intestine (31.8%) [1,2]. GISTs are considered to arise from interstitial cells of Cajal and are characterized by expression of tyrosine kinase receptor protein KIT (CD117)–the main diagnostic marker for these tumors [3]. Activating mutations in *KIT* proto-oncogene and homologous *PDGFRA* receptors result in constitutive activation of these proteins and are crucial initiating events in GIST pathogenesis. Although the key aspects of GIST pathogenesis are already elucidated, the mechanisms underlying overexpression of oncogenes and the role of gene expression regulators, like non-coding RNAs, in development of GISTs are not well investigated, yet.

Long intergenic non-coding RNAs (lincRNAs) are long (>200 nucleotides) non-coding RNAs that do not overlap exons of protein coding genes and comprise more than half of human long non-coding RNAs (lncRNAs) [4]. In recent years these non-coding RNAs emerged as important regulators of multiple cellular and molecular processes such as alternative splicing, chromatin modifications, transcriptional and post-transcriptional regulation, mRNA activity, stability and degradation and were found to be implicated in pathogenesis of diseases and development of cancer [4,5]. For example, the extensively studied lincRNA metastasis-associated lung adenocarcinoma transcript 1 *(MALAT1*) is overexpressed in lung adenocarcinoma, breast, pancreatic, colon, prostate, and hepatocellular carcinomas while promoting cell proliferation and metastasis [5,6]. Another lincRNA, Imprinted Maternally Expressed Transcript *H19*, has been shown to act both as tumor suppressor and oncogene in different types of cancer, playing an important role in epithelial mesenchymal transition and tumor growth and is a precursor of oncogenic microRNA miR-675 [7–10]. LncRNAs can also interact with small non-coding RNAs, i.e. acting as competing endogenous RNAs and sponging microRNAs through their binding sites [11]. In this way, lncRNAs can suppress microRNAs from binding to their targets and modulate target gene expression or be regulated by microRNAs.

Up to now, only two lncRNAs—*HOTAIR* and *CCDC26*—were investigated in GIST in more detail and were associated with high risk grade, metastasis and sensitivity to a drug imatinib [12–14]. Therefore, in this study we aimed to investigate lincRNAs deregulated in GIST and find new GIST-lincRNA associations. We performed next-generation sequencing data analysis and validated expression of three deregulated and cancer associated lincRNAs–*MALAT1*, *H19* and *FENDRR*–in GIST and adjacent tissues. Further analysis revealed correlations between overexpressed investigated lincRNAs in GIST tissue and genes involved in GIST pathogenesis, as well as with GIST deregulated microRNAs.

## Materials and methods

### Clinical samples, RNA material, gene expression and sequencing data

Paired formalin-fixed, paraffin-embedded (FFPE) tumor and adjacent tissue samples from 37 patients (total 74 samples) diagnosed with GIST of gastric origin were included in this study. The study group consisted of 22 women and 15 men with the average age of 66.95 (SD ± 12.39). GIST diagnosis was based on GIST morphology and positive KIT (CD117) immunohistochemical staining. Risk grade of tumors was assessed according to National Institutes of Health (NIH) GIST Consensus Criteria [15]. GIST patients included in the study were classified as: 7 high risk grade, 11 moderate, 15 low and 4 very low risk grade. Based on the GIST histological subtype, the majority of tumors in this study were of spindle cell type (n = 26), with several cases of epithelioid (n = 7) and mixed spindle and epithelioid (n = 4) cell type. *KIT* and *PDGFRA* mutational status was verified for 32 patients: 18 patients had *KIT* gene mutations exon 11 (n = 17), and exon 9 (n = 1), 9 patients had *PDGFRA* gene mutations in exon 18 (n = 6) and exon 12 (n = 3), while 5 patients were *KIT/PDGFRA* wild type. Distribution of all evaluated parameters did not differ in discovery and validation cohorts (p-value > 0.05). Fisher’s exact test was used for qualitative measurements and *t*-test was used for quantitative measurements.

RNA and DNA material was extracted from FFPE tissue samples using commercial miRNeasy FFPE and Qiamp FFPE DNA tissue Kits (Qiagen) following manufacturers recommendations.

Small RNA sequencing data (HiSeq2500, Illumina) of paired samples from 15 GIST patients was used for primary detection of deregulated lincRNAs (discovery cohort), while validation cohort for lincRNA expression analysis consisted of 22 patients’ paired samples. RNA libraries were prepared for sequencing using TruSeq Small RNA Sample Preparation Kit (Illumina).

More detailed characteristics of patients, GIST mutational status, RNA/DNA extraction, library preparation and NGS sequencing methods, as well as detailed methodology of *KIT*, *PDGFRA*, *ETV1* and microRNA gene expression analysis were described in our previous publication [16]. Small RNA sequencing data can be accessed at NCBI’s Gene Expression Omnibus (series accession number GSE89051).

The study was approved by the Kaunas Regional Biomedical Research Ethics Committee (No. BE-2–8). All patients have signed an informed consent form to participate in the study.

### lincRNA sequencing data analysis

Sequencing data analysis was performed using web-based tool miRMaster [17], where preprocessed sequencing reads (after adapter trimming, quality filtering and read collapsing) were mapped against the NONCODE 2016 database [18] of lncRNAs, using default parameters. P-values were generated by Kruskal-Wallis test and used for evaluation of gene expression changes after Benjamini-Hochberg adjustment for multiple testing. Changes with an adjusted probability value below 0.05 were considered as significant.

### Validation of lincRNA expression by reverse transcription quantitative real-time PCR (RT-qPCR)

Paired RNA samples of 22 GIST patients (total 44 samples) were included in the lincRNA expression validation cohort. RNA was reverse transcribed using High-Capacity cDNA Reverse Transcription Kit (Thermo Fisher Scientific). Expression of lincRNAs *MALAT1*, *H19* and *FENDRR* in GIST tumor and adjacent tissues was measured using commercial TaqMan Gene Expression Assays (Hs00273907_s1 for *MALAT1*; Hs00399294_g1 for *H19*; Hs05044154_s1 for *FENDRR*) and TaqMan Universal PCR Master Mix on the 7500 Fast Real-Time PCR system, according to the manufacturer’s protocol. Gene expression data was normalized to the expression levels of *GAPDH* housekeeping gene (Hs99999905_m1) and analyzed using comparative CT method.

### Statistical analysis

Differences between means of lincRNA expression validation data were analyzed using paired Student’s t-test. Expression changes with a Bonferroni adjusted p-values lower than 0.05 were considered significant. Spearman’s correlation analysis was applied on log_2_(2^-dCt^) values. Correlation coefficient values were assigned to groups from “negligible correlation” to “very high positive (negative) correlation” according to Mukaka M. [19]. LincRNA-oncogene and lincRNA-microRNA expression correlations were considered significant with a p-value lower than 0.01.

Kruskal-Wallis multiple comparisons test was applied to investigate differences of lincRNA expression in different GIST phenotypes. P-values lower than 0.05 were considered significant.

All statistical analyses were performed using the statistical computing environment R (version 3.4.4) [20].

## Results

### LincRNA analysis using NGS data

Sequences were mapped to a total 7240 lincRNA transcripts from the NONCODE database. After application of selection criteria (Benjamini-Hochberg adjusted p-value < 0.05, 2 < log_2_FC < -2), 23 lincRNA transcripts (9 unique lincRNAs) were significantly deregulated in GIST tissue compared to adjacent tissue, with six lincRNAs being upregulated and three—downregulated (Table 1).

**Table 1:** Significantly deregulated lincRNA transcripts in GIST vs. GIST adjacent tissue (paired samples of 15 patients) obtained from small RNA sequencing data.

Transcript	lincRNA	p-value	Benjamini-Hochberg adjusted p-value^a^	Fold Change, GIST vs. adjacent tissue	Log_2_(FC), GIST vs. GIST adjacent tissue
ENST00000447298.1	H19	1.13×10^−6^	0.002	69.2	6.1
ENST00000428066.5	H19	1.13×10^−6^	0.002	68.9	6.1
ENST00000442037.5	H19	1.42×10^−6^	0.002	61.2	5.9
ENST00000446406.5	H19	2.99×10^−6^	0.002	34.2	5.1
ENST00000414790.5	H19	2.99×10^−6^	0.002	34.1	5.1
ENST00000412788.5	H19	2.99×10^−6^	0.002	34.0	5.1
ENST00000431095.5	H19	3.66×10^−6^	0.002	33.9	5.1
ENST00000422826.1	H19	2.99×10^−6^	0.002	33.9	5.1
ENST00000411754.5	H19	3.66×10^−6^	0.002	33.8	5.1
ENST00000439725.5	H19	3.66×10^−6^	0.002	33.7	5.1
ENST00000417089.5	H19	3.66×10^−6^	0.002	33.7	5.1
ENST00000582452.1	LINC00908	4.99×10^−5^	0.016	33.3	5.1
ENST00000598996.2	FENDRR	2.20×10^−5^	0.011	15.8	4.0
ENST00000519898.5	CARMN	1.18×10^−4^	0.028	11.1	3.5
ENST00000610481.1	MALAT1	2.34×10^−4^	0.046	8.2	3.0
ENST00000544868.2	MALAT1	3.28×10^−5^	0.012	6.6	2.7
ENST00000508832.2	MALAT1	1.47×10^−4^	0.034	6.0	2.6
ENST00000534336.1	MALAT1	8.87×10^−5^	0.022	5.3	2.4
ENST00000619449.1	MALAT1	8.55×10^−5^	0.022	5.0	2.3
ENST00000637098.1	LINC00862	8.29×10^−5^	0.022	4.6	2.2
ENST00000582320.2	AC024267.1–201	6.26×10^−5^	0.019	0.1	-2.9
ENST00000607097.1	AC084346.2–201	4.58×10^−6^	0.002	0.1	-3.2
ENST00000413053.2	MIR194-2HG	3.67×10^−5^	0.013	0.01	-6.6

^a^ The difference is significant when Benjamini-Hochberg adjusted p-value is < 0.05.

FC–fold change

### Overexpression of lincRNAs *MALAT1*, *H19* and *FENDRR* verified in an independent group of GIST patients

**Fig 1:**
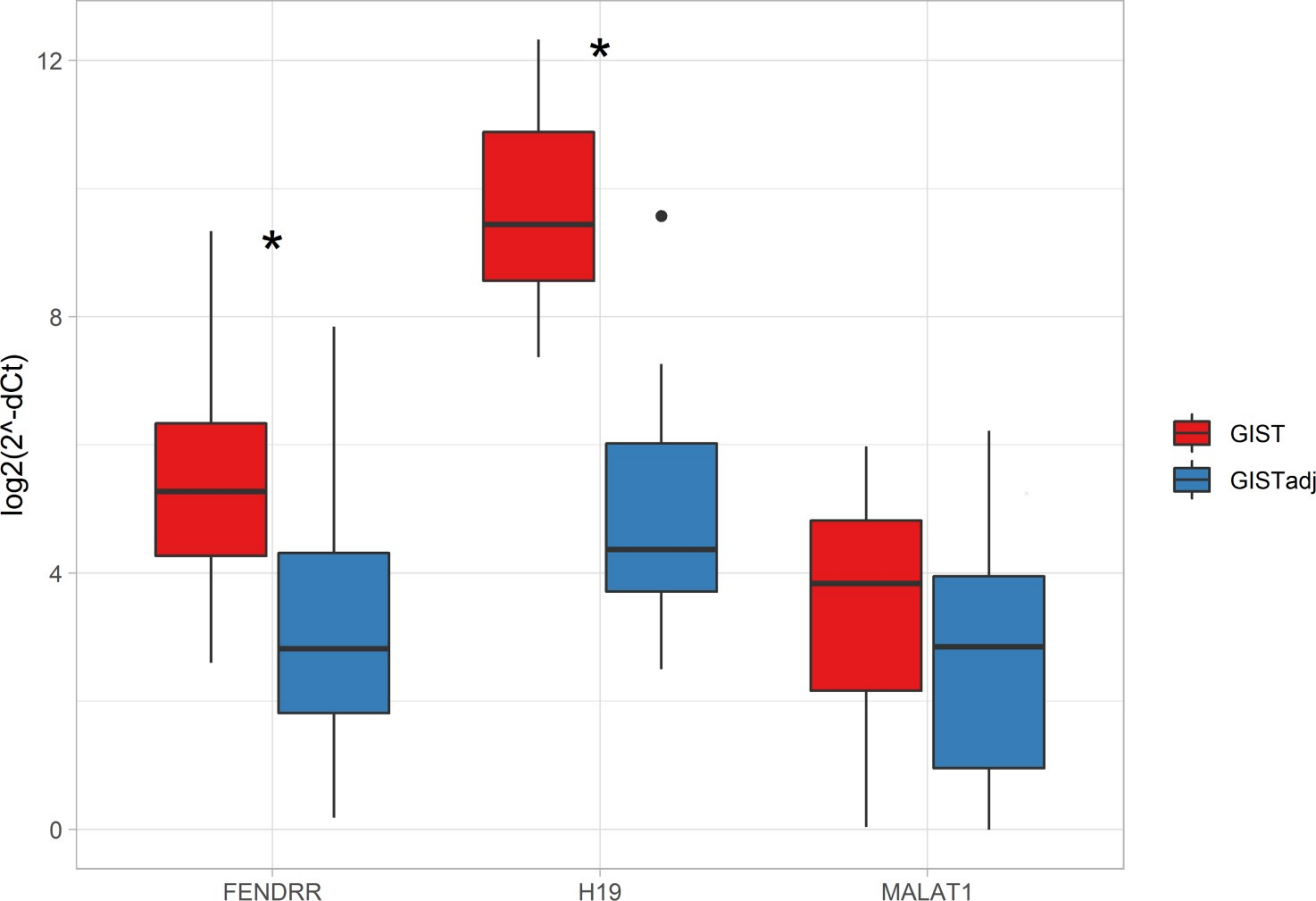
LincRNAs *FENDRR*, *H19* and *MALAT1* expression in GIST vs. adjacent tissue (paired samples of 22 patients). * p-value < 0.05 (Bonferroni adjusted p-value); whiskers–min. and max. values. LincRNA expression was normalized to expression levels of *GAPDH*.

Since the NGS analysis was based on small RNA sequencing, we further validated differential expression of lincRNAs in an independent group of 22 GIST patients (paired GIST and adjacent tissue samples), using RT-qPCR analysis. Three lincRNAs–*MALAT1*, *H19* and *FENDRR* shown to be significantly deregulated in GIST vs. adjacent tissue in our NGS data and previously associated with oncogenic processes, were selected for this validation step. Gene expression analysis results were in line with NGS data. All three investigated lincRNAs were upregulated in GIST tissue compared to adjacent tissue (Fig 1 and S1 Table). Significant upregulation was observed in expression levels of *H19* and *FENDRR*, with fold changes 25.8 (Bonferroni adjusted p-value = 3.70×10^−11^) and 4.7 (Bonferroni adjusted p-value = 4.66×10^−4^), respectively; while *MALAT1* was overexpressed nominally (1.7-fold, Bonferroni adjusted p-value = 0.141).

### Correlation analysis of *MALAT1*, *H19*, *FENDRR* and GIST associated oncogenes

**Fig 2:**
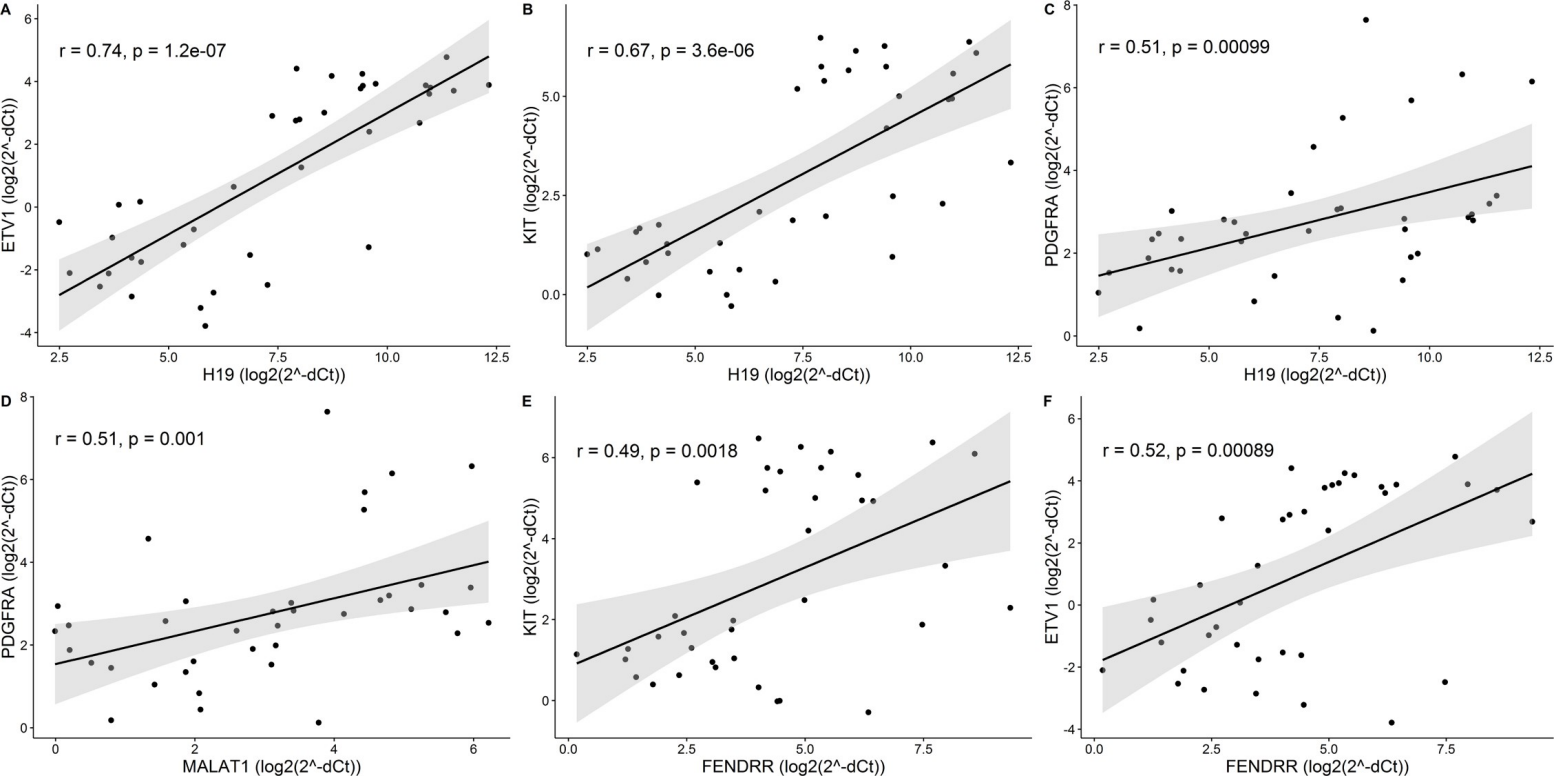
Significant correlations between expression levels of lincRNAs and GIST associated oncogenes *KIT*, *ETV1* and *PDGFRA*. (A) High positive correlation between *H19* and *ETV1*, (B-F) Moderate correlation scatter plots. r–Spearman’s rank correlation coefficient; correlation is significant when p < 0.01.

To examine potential effects of changes in lincRNAs expression on GIST pathogenesis, association analysis between lincRNAs and GIST associated oncogenes *KIT*, *PDGFRA* and *ETV1* was performed. Expression data of *KIT*, *PDGFRA* and *ETV1* has been previously described [16] and all three genes were shown to be significantly overexpressed in GIST tissue of our study group. High positive correlation was observed between expression levels of *ETV1* and *H19* (r = 0.74, p = 1.2×10^−7^) (Fig 2A, S2 and S3 Tables), while expression levels between *H19* and *KIT* or *PDGFRA*, *MALAT1* and *PDGFRA*, *FENDRR* and *KIT* or *ETV1* correlated moderately (r-values between ±0.5 and ±0.7, p-value < 0.01) (Fig 2B–2F, S2 and S3 Tables).

### Correlation analysis of *MALAT1*, *H19*, *FENDRR* and microRNAs differentially expressed in GIST

**Fig 3:**
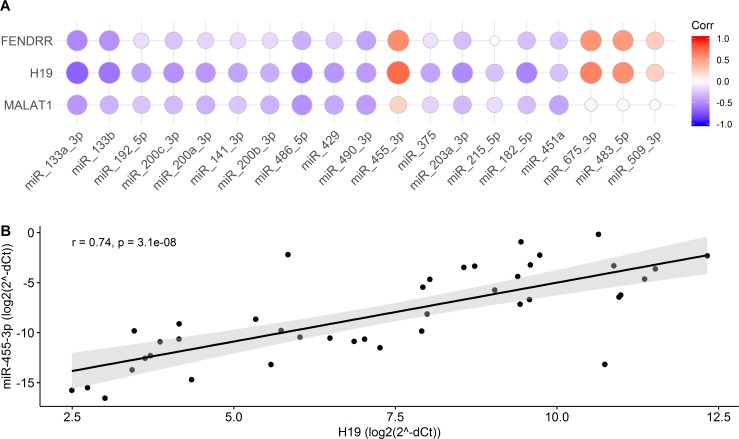
Correlation of lincRNAs and microRNAs. (A) Correlogram of GIST deregulated validated microRNAs and lincRNAs *FENDRR*, *H19* and *MALAT1*. The size and color intensity of the circle reflects the strength of Spearman correlation. (B) Significant high positive correlation between lincRNA *H19* and miR-455-3p. Spearman’s correlation coefficient and p value are shown in the plot.

Since it has been shown that lncRNAs can interact with other non-coding RNAs and participate in regulation of tumorigenic processes, lincRNA-microRNA correlation analysis was performed using expression data of 19 microRNAs, shown to be differentially expressed in GIST tissue compared to adjacent tissue in our previous study [16]. Spearman’s correlation test revealed high positive correlation between *H19* and miR-455-3p (r = 0.74, p = 3.1×10^−5^) (Fig 3B, S2 and S3 Tables), while expression of miR-133a-3p, miR-133b, miR-486-5p, miR-203a-3p, miR-182-5p, miR-675-3p, and miR-483-5p correlated moderately with expression levels of *H19* (r-values between ±0.5 and ±0.7, p-value < 0.01) (Fig 3A, S2 and S3 Tables). Significant moderate positive correlations were also observed between *FENDRR* and miR-455-3p, miR-675-3p, miR-483-5p (0.5 < r < 0.7, p-value < 0.01). *MALAT1* showed only low or no correlation with microRNAs differentially expressed in GIST (Fig 3A, S2 and S3 Tables).

### *MALAT1*, *H19*, *FENDRR* and GIST phenotype association analysis

To investigate possible clinical significance of validated lincRNAs in GIST (validation group n = 22 patients), subphenotype analysis based on histological types (spindle cell type (n = 14), epithelioid cell type (n = 4), mixed spindle and epithelioid cell type (n = 4)), disease risk grade (high (n = 3), moderate (n = 7), low (n = 9) and very low grade (n = 3)) and GIST mutational status (*KIT* mutant (n = 9), *PDGFRA* mutant (n = 6), *KIT/PDGFRA* wild type (n = 2)) was performed (S4 Table). However, multiple group comparison analyses did not reveal any significant differences in expression levels of *MALAT1*, *H19* or *FENDRR* between different histological subtypes, GIST risk grades or *KIT/PDGFRA* mutational status (S4 and S5 Tables).

## Discussion

Although a number of studies have elucidated the importance of lncRNAs in regulation of various biological processes and development of diseases, their profile and role in pathogenesis of GISTs are scarcely investigated. In the present study, we determined the profile of lincRNAs -a subset of lncRNAs–deregulated in GISTs. Further, we examined expression of three upregulated lincRNAs in a bigger group of independent patients and investigated associations with changes in expression of GIST-associated oncogenes and GIST-deregulated microRNAs.

Analysis of NGS data revealed 9 unique and strongly deregulated lincRNAs in gastric GIST tissue compared to adjacent gastric tissue. Among the top deregulated lincRNAs, three previously cancer associated lincRNAs—*MALAT1*, *H19* and *FENDRR*, were significantly upregulated in a discovery cohort. *MALAT1* and *H19* have been mostly described as oncogenic lincRNAs. Their overexpression, contributing to cell proliferation, metastasis, epithelial mesenchymal transition and poor prognosis, has been observed in esophageal squamous cell carcinoma, osteosarcoma, colorectal, gastric and other cancer types [6,8,21–25]. However, in our replication study only *H19* showed significant 25.8-fold overexpression in GIST tissue, while *MALAT1* was slightly overexpressed, but did not reach the required significance level. Both NGS and validation cohorts indicated *FENDRR* to be overexpressed in gastric GIST. Previous studies indicated *FENDRR* to be downregulated in a number of cancers, including osteosarcoma, gastric, breast, prostate cancers, and to be negatively associated with higher tumor stage, deeper invasion, shorter overall survival, suppression of apoptosis, promotion of cell proliferation and migration [26–29], but has been strongly overexpressed in an infantile hemangioma [30]. Furthermore, another study has shown that FENDRR was co-expressed with KIT–an oncogene crucial in GIST development–in a gallbladder cancer [31]. These findings are consistent with our results, where FENDRR expression significantly correlated with expression of KIT, and could explain the strong overexpression of FENDRR in GIST. However, the mechanisms of possible interactions between FENDRR and KIT have not been described yet and more thorough examination is needed in order to determine the role of these oncogenic molecules.

LincRNAs may affect expression of the coding genes through a variety of mechanisms such as regulation of chromatin, scaffolding RNA and proteins or acting as protein and RNA decoys [4]. Therefore, we tested possible correlations between differentially expressed lincRNAs and GIST-associated oncogenes. Analysis revealed a high positive correlation between expression levels of *H19* and *ETV1*. ETV1 is an ETS family transcription factor, which is highly expressed in all GISTs and is crucial for GIST growth and survival [32]. It has been shown that ETV1 is stabilized by another GIST-associated oncogene tyrosine kinase receptor KIT through the MAP kinase signaling pathway and they together form a positive feedback circuit to regulate GIST tumorigenesis [32,33]. Interestingly, another study revealed that *H19* might increase phosphorylation levels of components of MAP kinase pathway, such as pMEK and pERK, in colorectal cancer [34]. Moreover, a moderate, but significant correlation was observed between expression levels of *H19* and *KIT* or *PDGFRA* oncogenes in our study. This supports the theory of the positive feedback circuit between KIT and ETV1 [33], and the possible role of PDGFRA in stabilizing ETV1 [35]. These findings altogether suggest that *H19* might be an important factor in pathogenesis of GIST and is worth further investigation.

It has been reported that lncRNAs can interact with microRNAs by masking their targets’ binding sites, acting as competing endogenous RNAs and “sponge” microRNAs away from their mRNA targets, interacting with other microRNA related factors or being precursors for microRNAs [4,36]. Therefore, we used microRNA profiling data from our previous study [16] and evaluated their correlation with the expression of the three lincRNAs. A strong positive correlation was observed between expression levels of *H19* and miR-455-3p. Studies of this microRNA have demonstrated inconsistent results, where miR-455-3p has been shown to be overexpressed and/or have oncogenic properties in *KIT/PDGFRA* mutant GISTs and esophageal squamous cell carcinoma [37,38], but acted as a tumor suppressor in melanoma, pancreatic and non-small cell lung cancers [39–41]. Both *H19* and miR-455-3p were shown to be involved in the regulation of cancer related p53 signaling [42,43], which has been shown to be important in GIST oncogenesis, as well [44]. However, no direct association between *H19* and miR-455-3p could be found in literature. We also observed a moderate positive association between expression levels of oncogenic miR-675 and *H19*, as well as a moderate negative correlation between *H19* and miR-133 family members and other tumor suppressive microRNAs like miR-486-5p, miR-182-5p or miR-203a-3p [45–48]. MiR-675 is already known to be derived from *H19* [9,49] and has been shown to be implicated in the development of colorectal and breast cancers [50,51], while both miR-203a and *H19* have been shown to interact with E2F Transcription factor 1 (E2F1) [52–54]. This transcription factor is essential for cell proliferation [55] and can be upregulated by H19 in pancreatic ductal adenocarcinoma [54] or contrarily activates the promoter of H19 in breast cancer cells [53]. Furthermore, E2F1 binds to the promoter and activates miR-203a, while miR-203a can decrease expression of E2F1 through inhibition of CDK6, forming a feed-back loop [52]. Direct interactions between moderately correlated *H19* and miR-182, miR-486 or miR-133b have also been previously predicted from Argonaute-crosslinking and immunoprecipitation (AGO-CLIP) data [56] and requires additional experimental validation. As for the other identified correlations between *H19* or *FENDRR* and deregulated microRNAs no data has been found in the literature. Therefore, future studies are needed in order to confirm possible links between these oncogenic molecules and identify their interaction mode and possible role in oncogenesis.

We are aware that our study has certain limitations that need to be acknowledged. Firstly, due to the limited availability of fresh-frozen samples, FFPE samples were used for lincRNA and gene expression analyses in this study. Although, higher levels of RNA degradation can occur in FFPE tissues, previous studies have demonstrated that FFPE tissue can be a feasible material for gene expression analysis [57]. Secondly, we performed lincRNA profile analysis on small-RNA sequencing data. The purified RNA from FFPE is highly affected by hydrolysis and is fragmented into small (on average 100 nucleotide long) sequences [58–60]. These RNA fragments contain 5’-PO_4_ and 3’-OH end groups, which are used for selective ligation of sequencing adapters in Illumina Truseq small RNA sequencing protocol. Due to these features of FFPE-purified RNA (length and the end groups), small RNA-seq is sufficient to detect long non-coding RNAs in FFPE samples. However, to ensure the reliability of sequencing results, the findings were additionally validated by RT-qPCR method. We also admit that further functional experiments are needed to confirm the role of investigated lincRNAs in GISTs.

In summary, we performed lincRNA expression profile analysis in GISTs and confirmed significant overexpression of *H19* and *FENDRR* in GIST tissue compared to adjacent non-cancerous tissue. Association analyses revealed strong correlations between expression levels of lincRNA *H19* and GIST-related oncogene *ETV1* and cancer associated miR-455-3p. Despite the limitations described above, lincRNAs *MALAT1*, *H19* and *FENDRR* were not previously investigated in GISTs and we believe that our findings expand the knowledge in GISTs biology and elucidate possibly important components of GIST tumorigenesis for future studies.

## Supporting information

S1 Table LincRNA RT-qPCR data underlying Fig 1. (XLSX)

S2 Table MiRNA, lincRNA and mRNA expression data obtained from TLDA and RT-qPCR and used for Spearman correlation analyses. (XLSX)

S3 Table Results of Spearman correlation analysis between expression levels of investigated lincRNAs (*MALAT1*, *H19*, *FENDRR*) and GIST-deregulated miRNAs or GIST associated oncogenes. (XLSX)

S4 Table LincRNA qPCR data underlying subphenotype (histological type, risk grade and mutational status) analyses. (XLSX)

S5 Table Results of GIST subphenotype analysis. (XLSX)
